# Supplementary material for: Three small partner proteins facilitate the type VII-dependent secretion of an antibacterial nuclease
Source: mBio. 2023 Oct 10;14(5):e02100-23. doi: 10.1128/mbio.02100-23 (PMC10653861; doi:10.1128/mbio.02100-23)
Supplement: Supplemental figures and tables — Fig. S1, S2, S3, and S4; Tables S1, S2, and S3. [file mbio.02100-23-s0001.pdf]

Three small partner proteins facilitate the type VII-dependent secretion export of an antibacterial nuclease

Yaping Yang¶, Eleanor Boardman¶, Justin Deme, Felicity Alcock, Susan Lea and Tracy Palmer*

¶ These authors contributed equally

Tracy Palmer
Email: tracy.palmer@newcastle.ac.uk

Supporting Information

This PDF file includes:

Figures S1 to S4
Tables S1 to S3
SI References

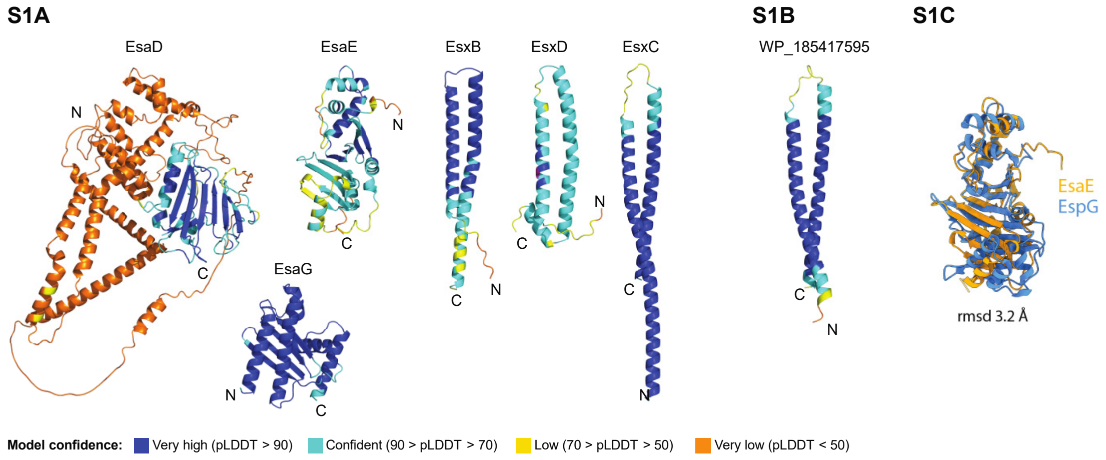

Fig. S1. Alphafold structures of EsaD and its accessory proteins

A. Alphafold models for each of the indicated proteins from *S. aureus* strain COL.

B. Alphafold model of WP_185417595, the EsxC protein from *Listeria booriae*, shown as an open pink arrow in Figure 1D. Models in (A) and (B) are colored according to the Alphafold model confidence.

C. Structural alignment of EsaE (modelled by Alphafold) (orange) with the crystal structure of EspG3 (PDB 4L4W). The aligned atoms have an RMSD of 3.2 Å.

S2A

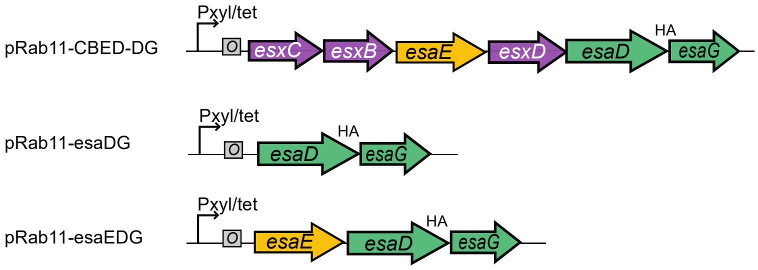

S2B

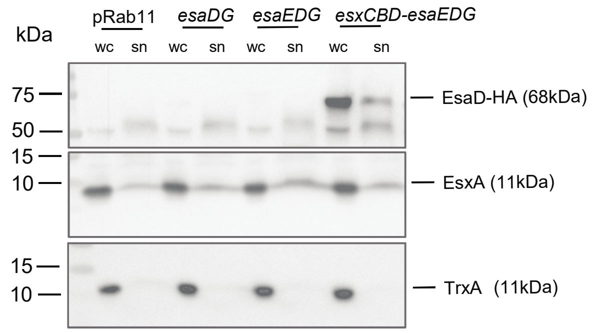

Fig. S2. Contribution of EsxBCD to EsaD secretion.

A. Schematic showing the constructs used in (B).

B. COL *ΔesxC-esaG* cells carrying empty vector (pRab11), or pRab11 carrying the indicated genes, were cultured in TSB growth medium. Following induction of plasmid-encoded gene expression, whole cell (wc) and culture supernatant (sn) fractions were isolated, prepared for immunoblot, and separated by SDS PAGE. EsaD was detected with antibodies against the C-terminal HA tag (upper panel). The T7-secreted control EsxA and cytoplasmic control TrxA were detected with antibodies against EsxA (middle panel) and TrxA (lower panel). N.B. the supernatant fractions analyzed here correspond to 6 x more culture volume than the cell fractions.

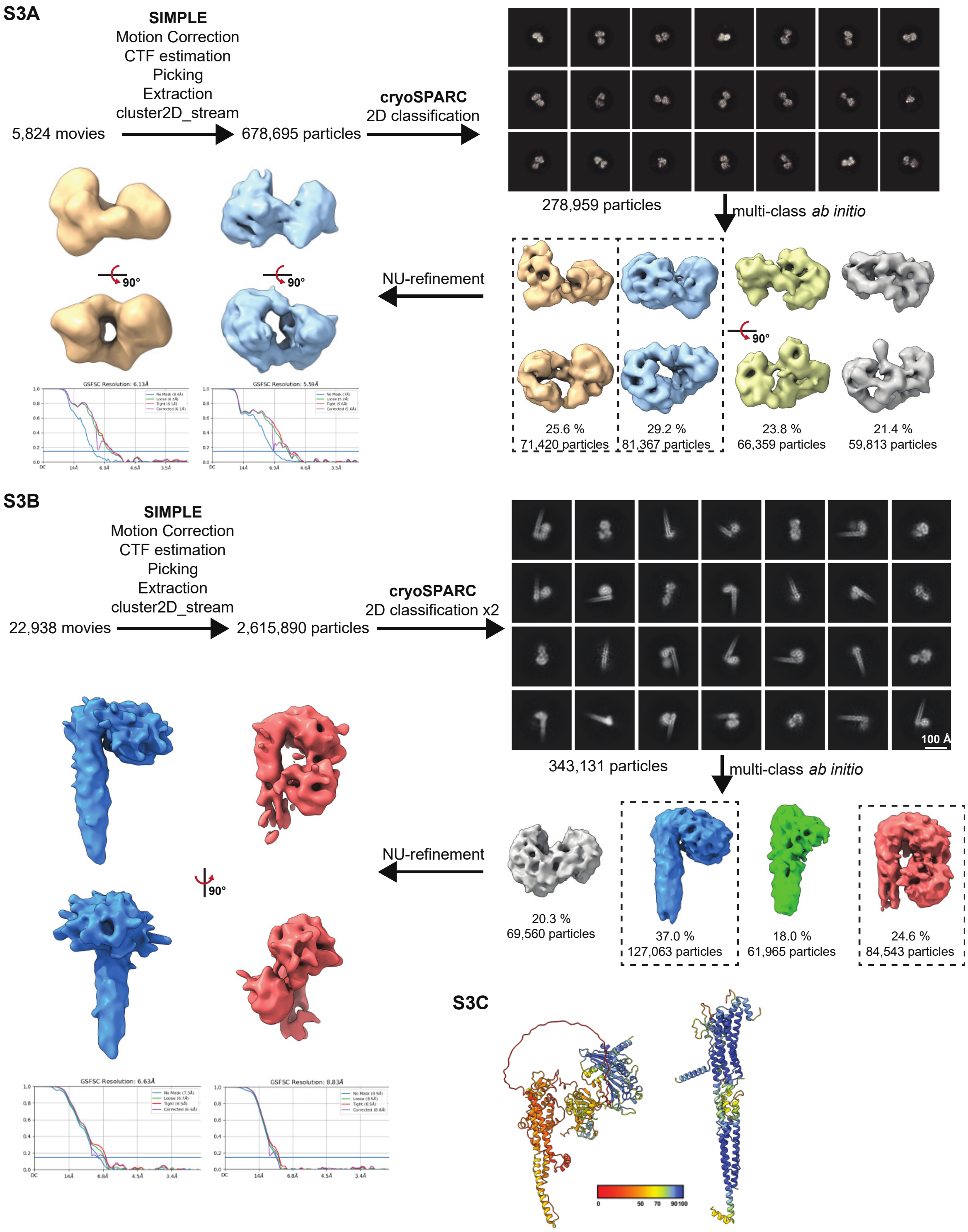

Fig. S3. Cryo-EM workflows

S3A. Cryo-EM image processing workflow for EsaDEG

S3B. Cryo-/EM image processing workflow for EsaDEG-EsxBCD

S3C. Alphafold models used for docking, colored by pLDDT score. Left, EsaDEG. Right, EsaD$_{1-220}$-EsxBCD.

S4

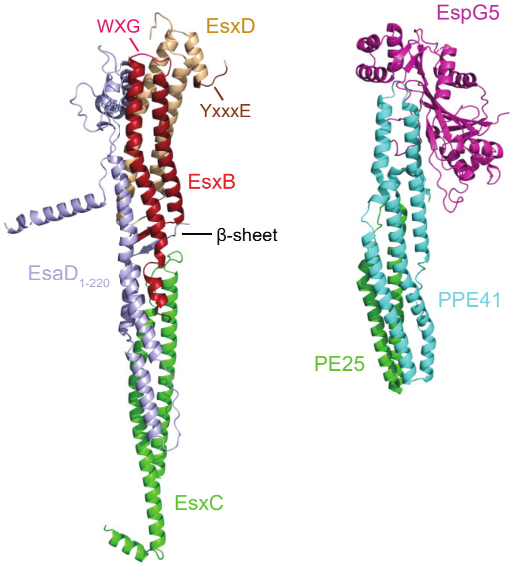

Fig. S4. Architecture of T7a and T7b substrate complexes.

An Alphafold model for $EsaD_{1-220}$-EsxBCD (left) alongside the crystal structure of a trimer of PE25, PPE41 and EspG3 from *M. tuberculosis* (right) (PDB 4W4L)(1).

Table S1. Strains used in this study

Strain	Genotype/description	Source/Reference
COL	*essC1 S. aureus* strain; MRSA *agr*	Reference (2)
COL Δ*esxC-esxG*	COL Δ*SACOL0277 – SACOL0282*	This study
COL Δ*essC*	COL Δ*SACOL0276*	Reference (3)
M15 (pREP4)	Commercial *E. coli* strain for expression from pQE plasmids. *F-, lac, ara, gal, mtl* [*KanR, lacI*]	Qiagen
JM110	*rpsL* (Str^r) *thr leu thi-1 lacY galK galT ara tonA tsx dam dcm supE44* Δ(*lac-proAB*) [F´ *traD36 proAB lacI*^{q}ZΔ*M15*]	Lab stock
DH5α	*Δ(argF-lac)169, φ80dlacZ58(M15), ΔphoA8, glnX44(AS), deoR481, rfbC1, gyrA96(NalR), recA1, endA1, thiE1 and hsdR17*	Lab stock

Table S2. Plasmids used in this study

Plasmid	Genotype/description	Source/Reference
pRab11	*E. coli* / *S. aureus* shuttle vector, $P_{xyl/tet}$ inducible promoter, cml^r, amp^r	Reference (4)
pRab11-CBED-DG	pRab11 carrying *esxC*, *esxB*, *esaE*, *esxD,* *esaD*^{H528A}*-HA*, *esaG* for tetracycline-inducible expression	This work
pRab11-CED-DG	pRab11-CBED-DG with *esxB* deleted	This work
pRab11-BED-DG	pRab11-CBED-DG with *esxC* deleted	This work
pRab11-CBE-DG	pRab11-CBED-DG with *esxD* deleted	This work
pRab11-CBD-DG	pRab11-CBED-DG with *esaE* deleted	This work
pRab11-esaDG	pRab11 carrying*, esaD*^{H528A}*-HA*, *esaG* for tetracycline-inducible expression	This work
pRab11-esaEDG	pRab11 carrying *esaE*, *esaD*^{H528A}*-HA*, *esaG* for tetracycline-inducible expression	This work
pIMAY-Z	*E. coli* / *S. aureus* shuttle vector, temperature sensitive, cml^r	Reference (5)
pIMAY_esxC-esaG	pIMAY-Z carrying *SACOL0277 – SACOL0282* deletion allele	This work
pQE70-GGED	pQE70 encoding two copies of SAOUHSC_00269 (EsaG), one each of SAOUHSC_00266 (EsaE) and SAOUHSC_00268 (EsaD) with a H528A mutation and a hexahistidine C-terminal tag. All genes codon optimised for *E. coli* expression.	This work
pREP4	*lacI KanR*	Qiagen
pQE70-GGED-his-CBED-tstrep	pQE70 carrying *esxC*, *esxB*, *esaE*, *esxD*-^{twinstrep}*,* *esaD*^{H528A}*-HA*, *esaG* for IPTG-inducible expression	This work
pQE70-GGED(1-477)-his-CBED	pQE70-GGED-his-CBED with EsaD toxin domain deleted	This work
pQE70-GGED(478-614)-his-CBED	pQE70-GGED-his-CBED with EsaD N-terminus deleted	This work

Table S3. Primers used in this study

Construct	Primer	sequence
pRab11-esaG	EsaG For	GGATTGGTACCAGGAGGTTTCTAGTT ATGACATTTGAAGAGAAGCTTAGCA
	EsaG Rev	GCTAAGAATTCTTATTCTTCTAGCTCTTTAATATAT
pRab11-esaDG	esaG_fwd	AGGAGGTTTCTAGTTATGAC
	esaG_rev	GGTACCATCATACTCTATCAATG
	EsaD_fwd	tgatagagtatgatggtaccaggaggtttctagttATGACAAAAGATAT TGAATATCTAAC
	EsaD_rev	gtcataactagaaacctcctctaagcgtaatctggaacatcgtatgggtaCTT ATTTAATATTCTTCTAATATTTCTTTCAC
pRab11-esaDG	H528	TGATGGAGGTGCCTTAATCGCTAG
(H528A substitution)	H528	TCGTCTGGTAATCTATCC
pQE70-GGED	codoptG1_fwd	ATTCATTAAAGAGGAGAAATTAAGCATGACATTTGAA GAAAAACTTAGC
	codoptG1_rev	ATTCATTAAAGAGGAGAAATTAAGCATGACATTTGAA GAAAAACTTAGC
	codoptG2_fwd	CTGGAAGAGTAATAAATTAAAGAGGAGAAATTAACCA TG
	codoptG2_rev	CTCCTCTTTAATGTTACTCTTCCAGCTCCTTAATG
	codoptE_fwd	CTGGAAGAGTAACATTAAAGAGGAGAAATTAAGC
	codoptE_rev	AATGGGCCCTTATTATTCTTCTGCCTTGTTG
	codoptDnostop_fwd	GGCAGAAGAATAATAAGGGCCCATTAAAGAG
	codoptDnostop_rev	AAGCTTAGTGATGGTGATGGTGATGCTTATTCAGAAT ACGACGG
	pQE70_GGEDco_fwd	CATCACCATCACCATCAC
	pQE70_GGEDco_rev	GCTTAATTTCTCCTCTTTAATG
	codoptH528A_fwd	GACGACGGTGGAGCCCTGATTGCTCGC
	codoptH528A_rev	GCGAGCAATCAGGGCTCCACCGTCGTC
pQE70-his-CBED-tstrep	QE backbone_rev	GCTTAATTTCTCCTCTTTAATG
	CBED-fwd	ttaaagaggagaaattaagcatgcatcaccatcaccatcacAATTTTAAT GATATTGAAACAATGGTTAAG
	CBED_rev	tggatctatcaacaggagtcctacagatcctcttctgagatgagtttttgttcTCC CTCAATATTATAGTAAAGC

pQE70-GGED (1-477)-CBED	9 delete 478-614_F	CATCACCATCACCATCAC
	9 delete 478-614_R	TGTATATTCAATATTTGCTTTAAG
pQE70-GGED-(421-614)-CBED	13Delete 1-420_F	ACCCATGGCCCAAAGGAT
	13Delete 1-420_R	CATGGTTAATTTCTCCTCTTTAATGG
pRab11-CBED-DG	1DG_fwd	AGGAGGTTTCTAGTTATGAC
	1DG_rev	GTACCATCATACTCTATCAATG
	CBED_F	ttgatagagtatgatggtacATGAATTTTAATGATATTGAAACAATG
	2CBED_R	gtcataactagaaacctcctCTATCCCTCAATATTATAGTAAAGC
pRab11-CED-DG	EsxB-deleted	tctagttATGAAAGATGTTAAGCGAATAG
	EsxB-deleted _R	aacctcctTTAATTCATTGCTTTATTAAAATATTC
pRab11-BED-DG	EsxC-deleted-F	ATGGGTGGATATAAAGGTATTAAAG
	EsxC-deleted-R	AACTAGAAACCTCCTGTAC
pRab11-CBE-DG	EsxD-4th-F	AGGAGGTTTCTAGTTATG
	EsxD-4th-R	TTACTCCTCTGCTTTATTAATATG
pRab11-CBD-DG	EsaE-deleted_F	tctagttATGACGTTGAGTGGAAAAATTAG
	EsaE-deleted_R	aacctcctTCATGGGTTCACCCTATC
pRab11-esaEDG	nnEDG_fwd	AGGAGGTTTCTAGTTATGAC
	nnEDG_rev	GTACCATCATACTCTATCAATG
	nnE_fwd	ttgatagagtatgatggtacaggaggtttctagttATGAAAGATGTTAAGCGAATAG
	nnE_Rev	gtcataactagaaacctcctTTACTCCTCTGCTTTATTAATATG
pIMAY-Z_CBEDDG	pIMAY-Z_fwd	CCGGGGGATCCACTAGTTC
	pIMAY-Z_rev	TCGATATCAAGCTTATCGATACCG
	up-700_fwd	atcgataagcttgatatcgaAGATGAAATTAAATATGAAGATTACAGAG

	up-700_rev	ttaagatagtAACATACCTCCCTCCTATTTAATTC
	down-700_fwd	gaggtatgttACTATCTTAATGTAAGACTAAACAATAAAG
	down-700_rev	agaactagtggatcccccggTTTTTCAATATATATAAATGAGCTCAAC

SI References

1. Ekiert DC, Cox JS. 2014. Structure of a PE-PPE-EspG complex from *Mycobacterium tuberculosis* reveals molecular specificity of ESX protein secretion. Proc. Natl. Acad. Sci. USA *111*, 14758-14763.

2. Dyke KG, Jevons MD, Parker MT. 1966. Penicillinase production and intrinsic resistance to penicillins in *Staphylococcus aures*. Lancet *1*, 835-838.

3. Ulhuq FR, Gomes MC, Duggan GM, Guo M, Mendonca C, Buchanan G, Chalmers JD, Cao Z, Kneuper H, Murdoch S, Thomson S, Strahl H, Trost M, Mostowy S, Palmer T. 2020. A membrane-depolarizing toxin substrate of the *Staphylococcus aureus* type VII secretion system mediates intraspecies competition. Proc. Natl. Acad. Sci. USA *117*, 20836-20847.

4. Helle L, Kull M, Mayer S, Marincola G, Zelder M-E, Goerke C, Wolz C, Bertram R. 2011. Vectors for improved Tet repressor-dependent gradual gene induction or silencing in *Staphylococcus aureus*. Microbiology *157*, 3314-3323.

5. Monk IR, Stinear TP. 2021. From cloning to mutant in 5 days: rapid allelic exchange in *Staphylococcus aureus*. Access Microbiol**.** *3*, 000193.
